# Clinical and Radiological Characteristics of Non-Benign Pineal Cyst Lesions in Children

**DOI:** 10.3389/fneur.2021.722696

**Published:** 2021-08-20

**Authors:** Ula Arkar, Rok Kučan, Mirjana Perković Benedik, Tadeja Hostnik, Tina Vipotnik Vesnaver, Tanja Loboda, Roman Bošnjak, Damjan Osredkar

**Affiliations:** ^1^Department of Pediatric Neurology, University Children's Hospital, University Medical Centre Ljubljana, Ljubljana, Slovenia; ^2^Department of Radiology, University Medical Centre Ljubljana, Ljubljana, Slovenia; ^3^Department of Neurosurgery, University Medical Centre Ljubljana, Ljubljana, Slovenia; ^4^Faculty of Medicine, Center for Developmental Neuroscience, University of Ljubljana, Ljubljana, Slovenia

**Keywords:** pineal cyst, child, pineal gland (the brain epiphysis), pineocytoma, headache, intracranial pressure, MRI, pineal cyst lesion

## Abstract

**Background:** With the increasing availability and advances in brain imaging, pineal cyst lesions (PCL) are becoming a common finding in the pediatric population. In the absence of evidence-based guidelines, optimal diagnostic and therapeutic approaches have not been established, and there is a risk of under- or overtreatment of these patients.

**Objectives:** The aim of our study was to evaluate the clinical presentation and radiological features of PCL in a cohort of pediatric patients and to identify clinical parameters more commonly associated with neoplasms in the pineal region. In addition, the prevalence of PCL in the pediatric population of Slovenia was estimated.

**Methods:** In this observational, cohort study, children treated at University Children's Hospital, Ljubljana, Slovenia in the period 1997–2016 were included if PCL was found on brain imaging. We analyzed indications for referral to a neurologist, clinical signs and symptoms, radiological features, treatment and outcome.

**Results:** The cohort consisted of 143 children with PCL. Pineocytoma was suspected in 31 children (21.7%). Six children underwent surgery – pineocytoma was confirmed in two cases and germinoma in one (2/3 of these children had signs of increased intracranial pressure (ICP), while PCL was benign in the remaining 4 cases. Only 2 PCL enlarged during the study period, both <2mm, none of these children developed neoplasm. Two children had PCL >20mm in diameter; both showed signs of increased ICP, one patient was found to have a germinoma of the pineal region, while the other had no neoplasm.

**Conclusions:** Most PCL do not change their features during radiological follow-up and even atypical PCL are very rarely associated with a malignant neoplasm of the pineal region. A PCL larger than 20 mm and signs of increased ICP were identified as potential markers for selecting patients at risk.

## Introduction

The primary function of the pineal gland is to regulate circadian and seasonal rhythms through melatonin secretion, but it is also involved in the onset of puberty and the activity of the sex glands (1). Pineal cystic lesions (PCL) are small lesions that are usually discovered incidentally by brain imaging (1, 2). In adults, the estimated prevalence ranges from 1.0 to 4.3% (3) and is slightly higher in women (4–7). In children, PCL occur on 1.9% of intracranial images (2, 3). PCL are often asymptomatic (2, 4), but may present with neurological symptoms and signs such as headache, seizures, dizziness, visual disturbances, hemiparesis (2, 4) and sleep disturbances (8). In some patients, PCL can exert a mass effect on surrounding tissues, causing Parinaud's syndrome, obstructive hydrocephalus or signs of increased intracranial pressure (ICP) (1, 5). Apart from these rare symptoms, the association between symptoms and PCL is extremely difficult to prove (7).

Magnetic resonance imaging (MRI) of the brain with gadolinium contrast enhancement is the method of choice for evaluation and follow-up of PCL (4). The distinction between simple and atypical PCL is important because neoplasms may arise in the pineal region. Simple PCL are round or oval, no larger than 20 mm in diameter, and surrounded by a uniform wall thinner than 2 mm that can be contrast-enhanced on MRI. Atypical PCL are usually larger, multicystic or septated, have a wall of variable thickness that may exceed 2 mm, and are by definition inhomogeneous, with various inclusions such as hemosiderin deposits or calcifications (9, 10). Atypical PCL are more often malignant, whereas simple PCL are more often benign glial cysts (4, 9). In adults, tumors of the pineal gland are extremely rare, whereas in children they account for 3–8% of all brain tumors (1). They are divided into germinal and non-germinal tumors, of which pineocytoma is the most common (1, 11). Despite advances in MRI, differentiating a benign PCL from a malignant tumor of the pineal gland can be challenging, and an optimal follow-up protocol is not clear (4, 11).

The aim of our study was to examine a cohort of children with PCL to identify clinical parameters more commonly associated with neoplasm in the pineal region.

## Materials and Methods

In this retrospective observational study medical records of children, diagnosed with PCL and treated at the University Children's Hospital of Ljubljana between 1.1.1997 and 1.8.2016 were evaluated. Patient data were collected from an electronic medical record system and from paper medical record archives. Medical records were evaluated with focus on age at first examination by neurologist/at the time of confirmed diagnosis, indication for referral to neurologist, clinical presentation, radiological characteristics of PCL (size, growth etc.), treatment, outcome, and frequency and duration of follow-up appointments.

Demographic data from the Statistical Office of the Republic of Slovenia (SORS) were used to estimate the prevalence of PCL in Slovenia.

MRIs were performed on 1.5-T Siemens Aera and 3T Siemens Magnetom Trio scanners. The standardized MR protocol included 3-mm sagittal and coronal T1-weighted spin-echo slices, centered on the pituitary region, 3-mm axial T2-weighted fast spin-echo slices of the entire head, and 1-mm sagittal T2-weighted 3D gradient-echo slices centered on the midline.

A chi-square test of independence was performed to examine the association between benign/non-benign PCL and ICP, size of PCL over 20 mm and focal neurological signs. The level of significance was set to *p* < 0.05.

The study was approved by Slovene National Ethics Committee (0120-472/2015-2).

## Results

### Basic Patient Data and Demographics

A cohort of 143 children was identified, of whom 83 were girls (58.0%). The mean age at first examination by a neurologist was 8.3 years (SD ±5.4 years; range, 1 day to 19.3 years). Imaging of the brain showing PCL for the first time was performed at a mean age of 9.4 years (SD ±5.4 years; range 1 day to 22.3 years). In six children in our cohort, radiological imaging was performed before the first appointment with a neurologist. Therefore, the time between the first appointment with a pediatric neurologist and a radiologically diagnosed PCL ranged from−2.3 to 10.2 years, median 3.3 months.

The estimated prevalence of pediatric PCL in Slovenia is 2.57/100,000 children, calculated based on SORS data as of 31.12.2015.

### Presentation and Medical History

The children in our cohort were referred to a neurologist for a variety of symptoms. The most common symptoms were headache (61; 42.7%), suspected epilepsy (17; 11.9%), epilepsy (9; 6.3%), developmental delay (7; 4.9%), head injury (5; 3.5%), and abnormal neurologic status (4; 2.8%). At the initial examination by a pediatric neurologist, the children presented with various neurological clinical signs and symptoms, which are shown in Table 1.

**Table 1:** Neurological signs and symptoms, reported at the first examination by a neurologist.

**Neurological sign or symptom**	**Number and percentage of children**
Headache	63 (45.3%)
Epileptic seizures	25 (18.0%)
Coordination disorders	13 (9.4%)
Vertigo	12 (8.6%)
Vision disorder	9 (6.5%)
Hemi-symptoms	5 (3.6%)
Cerebral nerve palsy	5 (3.6%)
Hypotonia	5 (3.6%)
Slow psychomotor development	5 (3.6%)
Torticollis	4 (2.9%)
Signs of elevated intracranial pressure	3 (2.2%)
Insomnia	3 (2.2%)
Syncope	3 (2.2%)
Sensory disorders	3 (2.2%)
Collapse	2 (1.4%)

^*^Other signs and symptoms included: balance disturbance, aggression, convulsive cry, convulsions, swallowing disorder, tics, elective mutism, anosmia, excessive fatigue, leg atrophy and lower back pain. These signs and symptoms were reported only in one child each.

Because headache was the most common symptom, we were interested in what type of headache was most common in children with PCL. We classified the headache types according to the criteria of International Headache Society. Of the 63 children, who reported headaches, 27 (42.9%) had migraine, 22 (34.9%) had an unspecified type, 12 (19.0%) had tension headaches, and two (0.03%) had a mixed headache type.

Of the 9 children who reported visual disturbances, three reported double vision, two reported anisocoria, one presented with optic nerve swelling (papilledema), one reported blurred vision, one presented with 6th cranial nerve palsy and one child reported episodes of amaurosis fugax.

Three children in our cohort had a sleep disorder, and all three suffered from insomnia. One boy underwent actimetry and one girl underwent continuous nocturnal monitoring of cardiorespiratory function. The tests showed no abnormal activity.

Three children in our cohort had endocrine disorders – one girl had signs of premature puberty, one girl had a menstrual cycle disorder and one boy had signs of diabetes insipidus. Both girls had normal levels of follicle-stimulating hormone (FSH), luteinizing hormone (LH) and estradiol in blood.

Family history for PCL was positive in two patients who were brothers, undocumented in two patients and negative in all others (97.2%). However, siblings of children with PCL were not actively screened for PCL, so more children with PCL may have had a positive family history.

Some patients had a history of other diseases. The most common were epilepsy (21; 14.7%), allergy (5; 3.5%), prematurity (4; 2.8%), brain tumor outside the pineal region (2; 1.4%), lactose intolerance (2; 1.4%), secondary amenorrhea (2; 1.4%), essential hypertension (2; 1.4%), and Asperger syndrome (2; 1.4%). Other conditions occurred in only one patient each. In our cohort, 62.2% had no other diseases.

After initial examination by a neurologist, 64 (44.76%) children were referred to an ophthalmologist. The most common indications were headache (36; 56.3%), light sensitivity (7; 10.9%), visual field loss (5; 7.8%), double vision (4; 6.3%), anisocoria (3; 4.7%) and vertigo (2; 3.1%). Ophthalmological examination was normal in 40 children (62.5%). Visual acuity defects were noted in 9 (14.1%), papilledema in 4 (6.3%), bulbomotor dysfunction in 3 (4.7%), and visual field defects in 3 children (4.7%). Visual evoked potential (VEP) was tested in four children: only one boy showed abnormal asymmetric signals.

A chi-square test of independence was performed to examine the relation between PCL type (benign vs. non-benign) and focal neurological signs. There was no significant retaionship between PCL type and the presence of focal neurological signs (*p* = 0.19).

### Radiologic Features of PCL

All children had at least one MRI of the head. In 128 children (89.5%), MRI was the first imaging examination, and in the remaining 15 children (10.5%) a Computed Tomography (CT) scan of the head was performed first. Gadolinium contrast was used in 80 of 143 children (55.9%). Simple PCL were seen in 90 children (62.9%), while atypical PCL were seen in 46 (32.2%). In seven children (4.9%), we could not evaluate the cysts as simple or atypical because we did not have access to their MR images.

**Figure 1:**
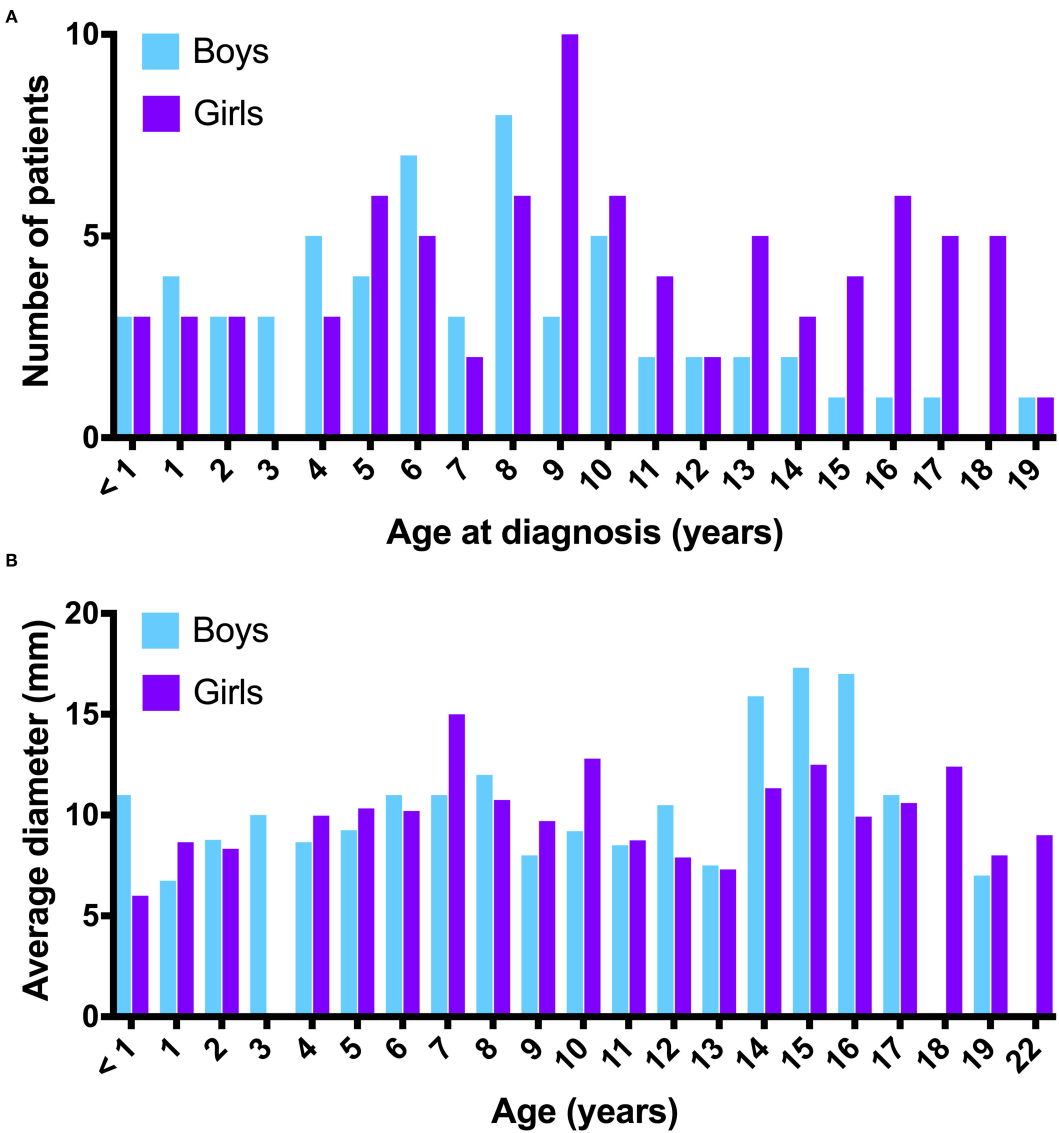
**(A)** Number of patients with a PCL distributed by age at diagnosis (years) and sex. **(B)** Distribution of average PCL diameters in the cranio-caudal direction by age (years) and sex.

**Figure 2:**
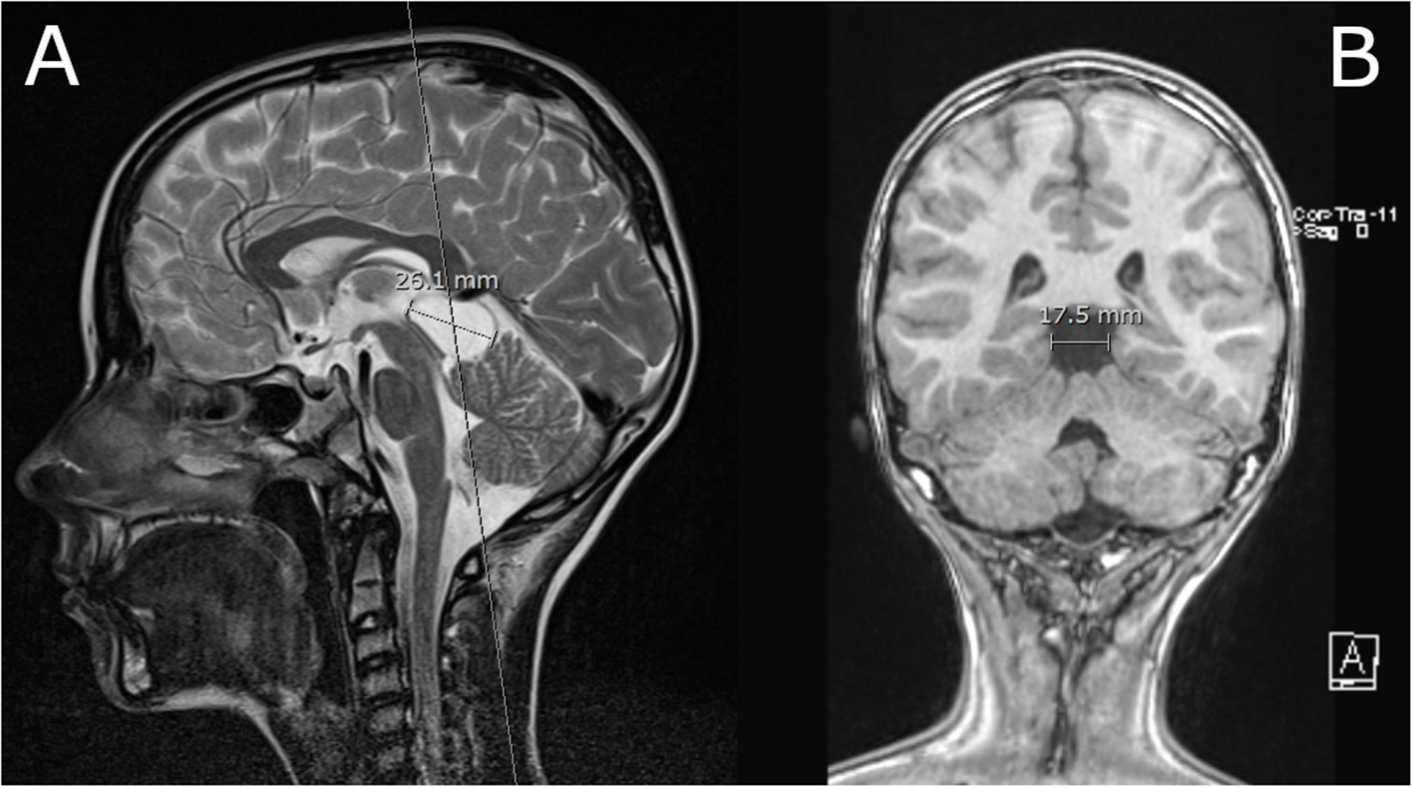
**(A)** T2 weighted image in mid-sagital plane; Large PCL that slightly displaces tectal plate and vermis. The aqueduct is patent, there is no evidence of obstructive hydrocephalus. **(B)** T1 weighted image of the PCL in the coronal plane.

The diameter of the PCL in the sagittal plane in the cranio-caudal direction averaged 10.2 mm (SD ±4.2 mm; range 2.5–30 mm), and in the antero-posterior direction averaged 8.6 mm (SD ±3.5 mm; range 2–30 mm). The distribution of the average diameter of the PCL in the cranio-caudal direction stratified by different age groups and sex is shown in Figure 1. An example of a large PCL seen on MRI is shown in Figure 2.

Of all 143 patients, 2 patients had pineocytoma and one had germinoma of the pineal region. Radiological imaging and laboratory investigations were also done in all children with suspected tumors. Laboratory tests of blood were performed in 26 (83.9%) children, MRI with contrast administration in 15 (48.4%) children, laboratory tests of cerebrospinal fluid (CSF) in 8 (25.8%) children, and MRI of the spinal canal in 2 (6.5%) children. Blood tests included tumor markers such as human chorionic gonadotropin β, α-fetoprotein, neuron-specific enolase, and others. One patient with suspected pineocytoma had pleocytosis of the CSF.

Signs of increased ICP were noted in 5 children. In 2 children, it was probably a transient CSF flow obstruction, as the symptoms disappeared with time, while the remaining 3 children were treated surgically. One patient had an astrocytoma outside the pineal region, the other had a germinoma of the pineal region, and the third had a pineocytoma. In both neoplasms of the pineal region, the increased ICP was due to pressure of the neoplasm on the aqueduct. A chi-square test of independence was performed to examine the relation between PCL type (benign vs. non-benign) and ICP. Increased ICP was significantly related to non-benign PCL (*p* < 0.001).

Two children had PCL with a diameter >20mm. Both presented with signs of increased ICP. One patient presented with headache, nausea and vomiting. He had an atypical cyst measuring 26 × 17 mm on MRI. An arachnoid cyst was suspected but not confirmed as the boy was treated conservatively. The other patient presented with vomiting. He had a 30 x 30 mm PCL on CT. He was treated with surgery – a germinoma of the pineal region was confirmed. A chi-square test of independence was performed to examine the relation between PCL type (benign vs. non-benign) and the PCL size (below vs. over 20 mm). A PCL size over 20 mm was significantly related to non-benign PCL (*p* < 0.001).

### Treatment and Follow-Up

In our cohort, 38 children (26.6%) were referred to a neurosurgeon, of whom nine were treated surgically; 3/9 were operated on for a brain tumor outside the pineal region, one child had an astrocytoma of the insular region, one child had a choroid plexus papilloma, and one child had a tumor of the pituitary gland. Two of these patients had simple PCL of 9 mm and 5 mm, and one had atypical PCL of 14 mm, respectively. The clinical and radiological findings of the remaining 6/9 surgically treated patients are shown in Table 2.

**Table 2:** Clinical and radiological findings in surgically treated patients with PC.

**Pt**	**Sex**	**Age at diagnosis (years)**	**Indication for imaging**	**↑ICP**	**NS**	**Type of PC**	**PC size (mm)**	**PC wall**	**Approach**	**Complications**	**HD**	**Outcome**
1	M	1.0	Unspecified headache	Yes	BMD	Atypical	18 × 15	PT	Occipital interhemispheric transtentorial	CSF fistula, hygroma, macrocrania	Pineocytoma	CI
2	M	8.7	Vomiting	Yes	nsf.	CT^a^	30 × 30	N/A	Occipital transtentorial	Seizures after surgery; complications due to chemo- and radiotherapy (hypophyseal nanosomia, hypogonadotropic hypogonadism)	Germinoma	CI
3	F	9.2	Epilepsy	No	nsf.	Simple	10 × 10	TU	Occipital transtentorial	None	Pineocytoma	PS
4	F	5.2	Epilepsy	No	nsf.	Atypical	13 × 9.5	PT	Occipital transtentorial	None	benign PC	CI
5	F	9.2	Tension headache	No	nsf.	Atypical	12 × 10	TU	Occipital transtentorial	None	benign PC	PS
6	M	12.6	Unspecified headache	No	BMD	Atypical	15 × 12	PT	Occipital transtentorial	None	benign PC	PS

*M, male; F, female; ICP, intracranial pressure; N/A, not applicable; NS, neurologic status; BMD, bulbomotor disorder; nsf., no special features; PC, pineal cyst; PT, posteriorly thickened wall; TU, thin, uniform wall; HD, histologic diagnosis; CI, clinical improvement; PS, persistent symptoms*.

a *Differentiation between simple and atypical PC is not possible on CT scan*.

Occipital transtentorial access was used in 5/6 surgically treated patients from our cohort. In Pt. 1, occipital paramedian transtentorial approach was used. None of these patients underwent endoscopic surgery. Two patients experienced surgery-related complications. In Pt. 1, a CSF fistula developed after the surgery, resulting in the formation of a subdural hygroma and consequent increase in head circumference. The increased ICP due to the hygroma was treated with high dose corticosteroids, which caused adrenal inhibition. Pt. 2 suffered a postoperative seizure. He was also treated with post-operative chemotherapy and radiotherapy resulting in pituitary nanosomy and hypogonadothrophic hypogonadism.

Of the children operated on for PCL tumors, two patients had signs of increased ICP and presented with headache or vomiting; symptoms resolved after surgical treatment. In the third patient, who had epilepsy before surgery, symptoms persisted after surgery. Of the remaining 3/6 patients with benign but atypical PCL who were treated surgically, 1 patient showed resolution of their symptoms after surgery, whereas in 2 patients symptoms remained unchanged. None of these patients had an increased ICP before surgery.

Most children with PCL were followed up by a neurologist or neurosurgeon. In our cohort, clinical follow-up was performed in 128 (89.5%) and radiologic follow-up in 91 (63.6%) patients. The mean duration of clinical and radiological follow-up was 3.5 years (range, 1 month to 18.3 years) and 2.5 years (range, 5 days to 10.5 years), respectively. Most children had 5 neurological examinations (range, one to 18) and 2 follow-up MR examinations (range, one to 10) during this time.

During radiological follow-up, only 2 (2.2%) PCL enlarged. The first case was a boy with suspected pineocytoma due to brain imaging for a head injury. At the first follow-up, germinoma was suspected. After 11 months, the PCL enlarged from 17.3 × 10.6 mm to 18.0 × 11.3 mm, mainly in the area of wall thickening. The second case was a girl with tension headache and suspected pinealocytoma, in whom the first CT head scan and the first control MRI showed no changes, but later there was a worsening of clinical symptoms. An urgent CT scan was performed at this time and showed posterior thickening of the PCL wall. PCL hemorrhage with pineal apoplexy was suspected. Surgical treatment was not performed at this time and on final examination by a neurologist, the girl no longer reported headaches.

## Discussion

With the increasing availability of radiological diagnostic methods, incidental findings, such as PCL, became more common, leading to unnecessary clinical and radiological follow-up of patients (3, 6). The results of our study suggest that most PCL are benign, as only 3/143 (2.1%) of the patients in our cohort had a tumor in the pineal region. Nevertheless, it is imperative to identify children with neoplasms of the brain early and treat them in a timely manner.

Differentiating benign from malignant PCL based on clinical and radiological features is challenging. In this observational cohort study, we sought to identify clinical and radiological parameters that would help a clinician to identify patients who are at risk for neoplasm in the pineal region and require further medical evaluation/treatment. Due to the small number of patients with neoplasms in the pineal region, we were unable to determine parameters that would unequivocally identify such patients, however increased ICP was found in 2/3 patients with a neoplasm in the pineal region and in one patient with a PCL and a neoplasm outside the pineal region, which was found to be statistically significant. Therefore, a combination of PCL and increased ICP could identify patients at risk. In our cohort, two patients had PCL with a diameter >20mm. Both patients had signs of increased ICP and one of them was found to have a germinoma in the pineal region. However, none of the two patients in our cohort, who had an increase in size of PCL developed a neoplasm in the pineal region, which was also found to be statistically significant. This is partially consistent with the review by Choque-Velasquez et al. who reported that PCLs requiring surgical treatment appear to develop with increasing size over time and are subsequently related with increased ICP (12). A focal neurologic deficit (cranial nerve palsy) was present in 1/3 of children with a malignant neoplasm in the pineal region in our cohort, but the presence of focal neurological signs was not found to be significantly related with a malignant neoplasm of the pineal region. In our patient cohort, 32.2% of PCL had atypical features, but only one of these patients had a neoplasm of the pineal region. This finding suggests that atypical appearance of PCL on MRI is not uncommon, but is rarely by itself associated with neoplasms of the pineal region.

Natural history data suggest that PCL often develop and change in childhood and then regress in adulthood (13). We saw a similar trend in our patient cohort, in which the incidence of PCL peaked at age 8 years in boys and at age 9 years in girls.

By the age of 18 years, more than 90% of adolescents report having experienced headaches (14). Headaches were also the most common symptom for referral to a pediatric neurologist in our study (42.7% of all cases). Migraine and non-specific headaches were prevalent in our patient cohort, which is consistent with the study by Seifert et al. in which PCL was more commonly associated with migraine headache (15).

There is no consensus in the literature regarding follow-up, but most authors agree that both clinical and radiological follow-up are necessary in some patients (7). Based on the results of our study, we cannot make evidence-based recommendations regarding the frequency of clinical and radiological follow-up. Larger, prospective studies are needed to better understand the relationship between clinical and radiologic features of patients with PCL and their follow-up, and to develop optimal diagnostic and treatment protocols.

In our study, we identified 143 children with PCL and estimated the prevalence of PCL in the pediatric population of Slovenia to be 2.57/100,000 children. As we could not find similar published results, we cannot compare the prevalence in Slovenia with the prevalence in other countries. However, it is important to emphasize that we only included children treated at University Children's Hospital in Ljubljana. We probably overlooked patients diagnosed with PCL in other hospitals in Slovenia (although we estimate that this is a small proportion of PCL patients, as most are referred to us), as well as asymptomatic and undiagnosed patients, so the true prevalence of PCL in the pediatric population of Slovenia must be higher than that reported here.

Our study has clear limitations. The sample size was not large enough to make a clear distinction between benign and malignant PCL. Due to the retrospective nature of the study, we could not evaluate all clinical parameters of the children with PCL and clinical and radiological follow-up of our cohort was not performed systematically. The majority of children had a negative family history, suggesting that PCL is not more common in family members, but this was not systematically verified in our study by performing MRI in siblings of children with PCL.

In summary, only few PCL are non-benign lesions that require further investigation or treatment. Most PCL do not change their features during radiological follow-up and even atypical PCL are rarely associated with a malignant neoplasm of the pineal region. In our study, PCL larger than 20 mm and signs of increased ICP were identified as potential markers for selecting patients at risk.

## Data Availability Statement

The raw data supporting the conclusions of this article will be made available by the authors, without undue reservation.

## Ethics Statement

The studies involving human participants were reviewed and approved by Slovene National Ethics Committee (0120-472/2015-2). Written informed consent from the participants' legal guardian/next of kin was not required to participate in this study in accordance with the national legislation and the institutional requirements.

## Author Contributions

DO, MP, and RK contributed to conception and design of the study. RK, TH, and RB organized the database. RK, TH, and UA performed the statistical analysis. UA and TL wrote the first draft of the manuscript. UA, TV, and DO wrote sections of the manuscript. All authors contributed to manuscript revision, read, and approved the submitted version.

## Conflict of Interest

The authors declare that the research was conducted in the absence of any commercial or financial relationships that could be construed as a potential conflict of interest.

## Publisher's Note

All claims expressed in this article are solely those of the authors and do not necessarily represent those of their affiliated organizations, or those of the publisher, the editors and the reviewers. Any product that may be evaluated in this article, or claim that may be made by its manufacturer, is not guaranteed or endorsed by the publisher.

## Abbreviations

CIR-UMCL: Clinical Institute of Radiology of the University Medical Centre Ljubljana
CT: Computed tomography
ICP: Intracranial pressure
MRI: Magnetic resonance imaging
PCL: Pineal cyst lesion
SD: Standard deviation
SORS: Statistical Office of the Republic of Slovenia.

## References

[1] Bruno F Arrigoni F Maggialetti N Natella R Reginelli A Di Cesare E. Neuroimaging in emergency: a review of possible role of pineal gland disease. Gland Surg. (2019) 8:133–40. 10.21037/gs.2019.01.02 31183323 PMC6534760

[2] Al-Holou WN Maher CO Murasko KM Garton HJL. The natural history of pineal cysts in children and young adults: clinical article. J Neurosurg Pediatr. (2010) 5:162–6. 10.3171/2009.9.PEDS09297 20121364

[3] Al-Holou WN Garton HJL Muraszko KM Ibrahim M Maher CO. Prevalence of pineal cysts in children and young adults. J Neurosurg Pediatr. (2009) 4:230–6. 10.3171/2009.4.PEDS0951 19772406

[4] Starke RM Cappuzzo JM Erickson NJ Sherman JH. Pineal cysts and other pineal region malignancies: determining factors predictive of hydrocephalus and malignancy. J Neurosurg. (2017) 127:249–54. 10.3171/2016.8.JNS16220 27767399

[5] Májovský M Netuka D Beneš V. Conservative and surgical treatment of patients with pineal cysts: prospective case series of 110 patients. World Neurosurg. (2017) 105:199–205. 10.1016/j.wneu.2017.05.155 28583453

[6] Nevins EJ Das K Bhojak M Pinto RS Hoque MN Jenkinson MD. Incidental pineal cysts: is surveillance necessary? World Neurosurg. (2016) 90:96–102. 10.1016/j.wneu.2016.02.092 26944882

[7] Májovský M Netuka D Beneš V. Clinical management of pineal cysts: a worldwide online survey. Acta Neurochir. (2016) 158:663–9. 10.1007/s00701-016-2726-3 26897024

[8] DelRosso LM Martin K Bruni O Ferri R. Sleep disorders in children with incidental pineal cyst on mRI: a pilot study. Sleep Med. (2018) 48:127–30. 10.1016/j.sleep.2018.05.003 29906628

[9] Fakhran S Escott EJ. Pineocytoma mimicking a pineal cyst on imaging: true diagnostic dilemma or a case of incomplete imaging? Am J Neuroradiol. (2008) 29:159–63. 10.3174/ajnr.A0750 17925371 PMC8119086

[10] Barboriak DP Lee L Provenzale JM. Serial MR imaging of pineal cysts: implications for natural history and follow-up. Am J Roentgenol. (2001) 176:737–43. 10.2214/ajr.176.3.1760737 11222216

[11] Korogi Y Takahashi M Ushio Y. MRI of pineal region tumors. J Neuro-Oncol. (2001) 54:251–61. 10.1023/A:1012773727022 11767291

[12] Choque-Velasquez J Colasanti R Baluszek S Resendiz-Nieves J Muhammad S Ludtka C. Systematic review of pineal cysts surgery in pediatric patients. Childs Nerv Syst. (2020) 36:2927–38. 10.1007/s00381-020-04792-3 32691194 PMC7649165

[13] Al-Holou WN Terman SW Kilburg C Garton HJ Muraszko KM Chandler WF. Prevalence and natural history of pineal cysts in adults: clinical article. J Neurosurg. (2011) 115:1106–14. 10.3171/2011.6.JNS11506 21780858

[14] Barea LM Tannhauser M Rotta NT. An epidemiologic study of headache among children and adolescents of southern Brazil. Cephalalgia. (1996) 16:545–9. 10.1046/j.1468-2982.1996.1608545.x 8980856

[15] Seifert CL Woeller A Valet M Zimmer C Berthele A Tölle T. Headaches and pineal cyst: a case-control study. Headache. (2008) 48:448–52. 10.1111/j.1526-4610.2007.00965.x 18005138

